# Case report of a rare cause of secondary hypertension illustrating the importance of cardio-obstetric preconception counselling

**DOI:** 10.1093/ehjcr/ytae092

**Published:** 2024-02-20

**Authors:** Theo A Meister, Rodrigo Soria, Laura Bubulyte, Giancarlo Spano, Vladimir Makaloski, Luigi Raio, Emrush Rexhaj

**Affiliations:** Department of Cardiology and BioMedical Research, University Hospital of Bern, Freiburgstrasse 20, 3010 Bern, Switzerland; Department of Cardiology and BioMedical Research, University Hospital of Bern, Freiburgstrasse 20, 3010 Bern, Switzerland; Department of Cardiology and BioMedical Research, University Hospital of Bern, Freiburgstrasse 20, 3010 Bern, Switzerland; Department of Cardiology and BioMedical Research, University Hospital of Bern, Freiburgstrasse 20, 3010 Bern, Switzerland; Department of Vascular Surgery, University Hospital of Bern, Bern, Switzerland; Department of Obstetrics and Gynecology, University Hospital of Bern, Bern, Switzerland; Department of Cardiology and BioMedical Research, University Hospital of Bern, Freiburgstrasse 20, 3010 Bern, Switzerland

**Keywords:** Case report, Hypertension, Pregnancy, Middle aortic coarctation

## Abstract

**Background:**

Cardiovascular diseases represent a leading cause of maternal morbidity and mortality in industrialized countries. High blood pressure during pregnancy is a major driver of short- and long-term cardiovascular health in both mother and child. Screening and adequate treatment of elevated blood pressure before pregnancy significantly reduce mortality risk to mother and child.

**Case summary:**

A 30-year-old woman with *middle aortic coarctation* (*MAC*) previously treated with aortic stenting was referred to our cardio-obstetrics with plans to become pregnant. The clinical examination revealed severe hypertension with a significant blood pressure gradient between the upper and lower limbs. The patient underwent computed tomography angiography showing re-stenosis of the aorta. After the analysis of the benefit risk of all treatment options, percutaneous transluminal aortic in-stent re-stenting was performed. Following the intervention, blood pressure profile significantly improved but remained slightly elevated further necessitating the introduction of an antihypertensive therapy.

**Discussion:**

This clinical case condenses several challenges encountered in the management of hypertension in women who plan to become pregnant. Firstly, it emphasizes the fact that secondary causes of chronic hypertension, including MAC, do not have to be overlooked in childbearing age patient. Secondly, it illustrates the need for a multidisciplinary analysis of all available treatment options in view of a future pregnancy. Finally, it discusses the particular follow-up and potential complications in pregnant women with MAC and aortic stent.

**Learning points**

- When facing a hypertensive woman with plans to become pregnant, secondary causes of arterial hypertension should always be considered and ruled out before conception.
- Women with secondary causes of arterial hypertension and plans to become pregnant should be referred to cardio-obstetric preconception counselling to review treatment options and establish a follow-up strategy for future pregnancies.

## Introduction

Screening, improved maternal medical care, and timely interventions during pregnancy have significantly decreased maternal mortality and morbidity in the last century.^1^ However, similar to the non-pregnant population, cardiovascular diseases and hypertension in particular are still a leading cause of maternal mortality worldwide.^2^

Chronic or pre-existing hypertension in pregnancy (CHP) affects 8% of reproductive-aged women (age 20–44 years old),^3^ and the prevalence of CHP has increased more than 13-fold in less than four decades.^4^ Chronic hypertension in pregnancy is associated with increased maternal risk of mortality [odds ratio (OR): 1.7], myocardial infarction (OR: 3.4), and stroke (OR: 3.4). Moreover, the risk of pre-eclampsia is also increased (OR: 2.6) and is associated with foetal and neonatal morbidity and mortality as shown by an increased risk of stillbirth (OR: 1.7) or prematurity (OR: 1.3).^5^

Chronic hypertension in pregnancy is defined as a systolic blood pressure (SBP) of ≥140 mmHg and/or a diastolic blood pressure (DBP) of ≥90 mmHg measured before conception or prior to 20 weeks of gestation and persisting for more than 42 days of postpartum.^6^ Mild hypertension is defined as a SBP between 140 and 159 mmHg and/or a DBP between 90 and 109 mmHg; severe hypertension is defined as SBP ≥ 160 mmHg and/or DBP ≥ 110 mmHg.^6^ Of note, this definition differs from the European Society of Cardiology & European Society of Hypertension (ESC/ESH) grading of hypertension used in non-pregnant adults.^7^

The ESC guidelines for the management of cardiovascular diseases during pregnancy, and more recently, the American College of Obstetricians and Gynecologists and the Society for Maternal-Fetal Medicine, have adopted blood pressure (BP) values of ≥140/90 mmHg as the threshold for initiation or titration of medical therapy for CHP.^6,8,9^ This threshold is justified by the results of the 2022 Chronic Hypertension and Pregnancy (CHAP) trial, which showed that drug treatment reduced maternofoetal morbidity without decreasing foetal weight or increasing maternal or neonatal complications at SBP ≥ 140 mmHg or DBP ≥ 90 mmHg.^10^ Importantly, this study also highlighted that even moderate elevation of BP can have a deleterious effect on the mother and foetus.

In the present clinical case, we aim to illustrate the value of preconception screening and management of CHP. We also stress the fact that secondary causes of hypertension represent up to 10% of all cases of hypertension between the ages of 18 and 49 years, a range that includes most women of childbearing age.^11^ Nevertheless, diagnostic and evidence-based management is challenging because secondary hypertension is often caused by rare conditions, as illustrated in this case of *middle aortic coarctation* (MAC).

## Summary figure


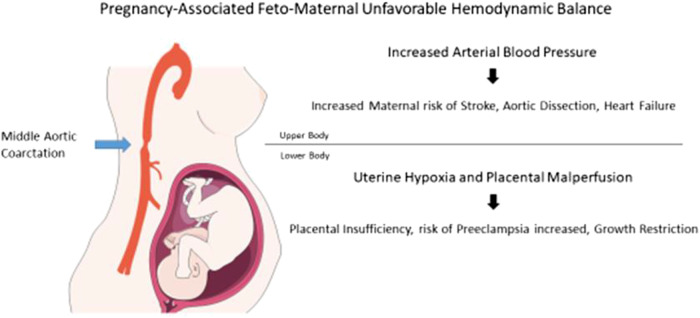


## Case presentation

We report the case of a 30-year-old woman previously diagnosed with a congenital supracoeliacal aortic stenosis. Twice in the last 7 years before presentation, she underwent percutaneous transluminal angioplasty (PTA) and aortic stenting with excellent clinical results. She was referred to our cardio-obstetric clinic due to a recent increase in SBP above 190 mmHg without treatment and plans to become pregnant.

She presented to us with complaints of leg paraesthesia that only occurred after sitting for long periods of time. She denied any claudication, abdominal angina, or other complaints.

Physical examination was remarkable for a body mass index of 33 kg/m^2^ and elevated BP in both arms (right arm 177/117 mmHg and left arm 180/121 mmHg). Four extremities BP measured in supine position revealed a maximal pressure gradient of 19 mmHg between the upper and lower limbs as well as pathological ankle–brachial indexes (ABI) bilaterally. (*Table 1*) The rest of the physical examination was unremarkable.

**Table 1:** Four extremities blood pressure measurement at rest, after exercise, and after re-stenting

	Right	ABI	Left	ABI
**At rest**				
A. brachialis	181 mmHg		189 mmHg	
A. tibialis posterior	170 mmHg	0.9	170 mmHg	0.9
A. dorsalis pedis	174 mmHg	0.92	170 mmHg	0.9
**After exercise**				
A. brachialis	181 mmHg		189 mmHg	
A. tibialis posterior	*NA*		175 mmHg	0.93
A. dorsalis pedis	167 mmHg	0.88	*NA*	
**After re-stenting**				
A. brachialis	150 mmHg			
A. tibialis posterior	170 mmHg	1.13	160 mmHg	
A. dorsalis pedis	165 mmHg	1.1	160 mmHg	1.07

ABI, ankle–brachial index.

A 24-h ambulatory BP measurement (24h-ABPM) confirmed the presence of severe hypertension (mean 24-h SBP of 157 mmHg and DBP of 120 mmHg). Transthoracic echocardiography showed normal left ventricular (LV) function (LV ejection fraction: 64%), with concentric LV remodelling but still normal LV muscle mass (indexed LV muscle mass: 66.5 g/m) and normal left atrium size (left atrial volume index: 27 m/m^2^). Echo Doppler of the abdominal aorta and renal arteries showed a supracoeliacal flow acceleration (from 100 to 360 cm/s during systole) compatible with a high-grade stenosis with turbulent flow and reduced resistance indexes in the renal arteries (resistance index: 0.39 right and 0.40 left).

**Figure 1:**
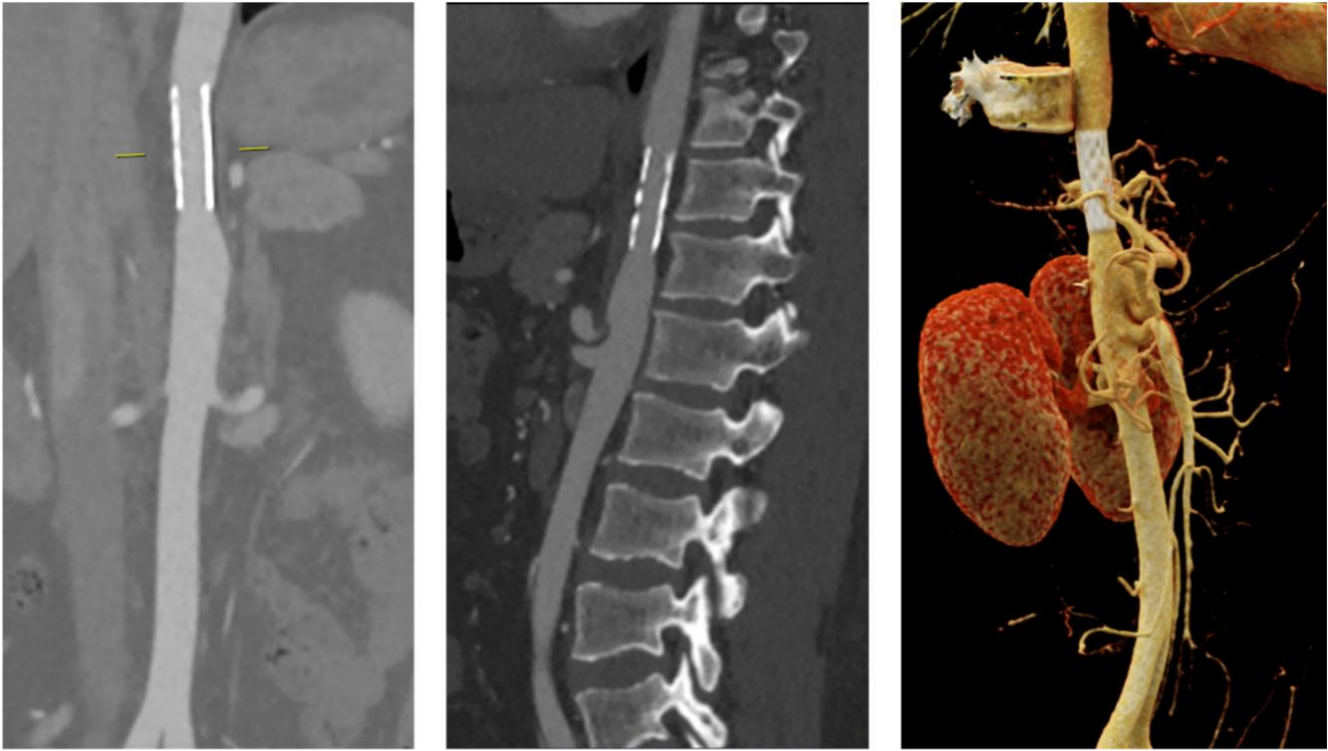
Computed tomographic angiography of the abdominal aorta. Coronal view (left), sagittal view (middle), and 3D reconstruction (right).

**Figure 2:**
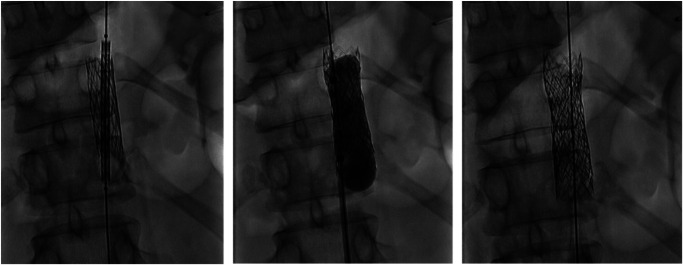
Fluoroscopy images of percutaneous transluminal angioplasty before (left), during (middle), and after (right) in-stent re-stenting.

A computed tomographic (CT) angiography of the aorta was performed showing an aortic stent in a supracoeliacal position without evidence of in-stent thrombosis. However, the inner diameter of the aorta cranial to the stent was normal (15 mm) but became significantly reduced (7 mm) at the level of the stent corresponding to 79% of in-stent re-stenosis (*Figure 1* and Supplementary material online). After discussion with the vascular surgeon, a repeat PTA was performed. The BP gradient measured invasively between the descending aorta and the right arm showed a difference of 15 mmHg. The patient underwent in-stent re-stenting (BeGraft Aortic Stent 16 mm × 58 mm) with a residual gradient of 9 mmHg after the intervention (*Figure 2*).

Four extremities BP measurements after the intervention showed a reduction of the BPs and a normalization of the ABI bilaterally (*Table 1*). A 24h-ABPM confirmed the reduction (mean 24-h SBP of 135 mmHg and DBP of 101 mmHg), but her BP profile remained significantly elevated with Grade 2 hypertension according to ESC/ESH 2018 guidelines.^7^ We started an antihypertensive treatment with Labetalol 200 mg b.i.d. with successful BP control (mean home BP values: 125/82 mmHg).

## Discussion

Coarctation of the aorta (CoA) is a segmental constriction of the aorta lumen that is usually located at the level of the isthmus near the aortic insertion of the *ligamentum arteriosum*. Coarctation of the aorta is a congenital heart disease with an incidence of 4 in 10 000 live births, which can be found in an isolated form or in association with other cardiac abnormalities or genetic syndromes. Although CoA is present in less than 1% of all patients with resistant arterial hypertension, it is the second most common cause of hypertension in children and young adults.^12^

Middle aortic coarctation also called middle aortic syndrome is a rare form of CoA (0.5–2% of all CoA) where the aortic constriction is located at the level of the lower thoracic and upper abdominal aorta with or without renal or mesenteric artery involvement. Congenital forms have been explained by the failure of the normal fusion of the two dorsal aortas and are occasionally associated with genetic syndromes like neurofibromatosis type 1, Alagille or Williams syndrome. Inflammatory or infectious diseases of the aorta can lead to acquired forms of MAC. The pathophysiology of hypertension in MAC is explained by renal hypo-perfusion leading to renin–angiotensin–aldosterone system activation (*renovascular hypertension*). Endothelial dysfunction and premature ageing are also important contributors to the elevated hypertension prevalence observed after CoA repair.^13^ It remains unknown if a similar mechanism could also play a role in MAC.

Clinically, MAC presents with severe arterial hypertension of the upper body, a systolic pressure gradient between the upper and lower limbs, and a systolic murmur audible in the abdomen or xiphoidal region. However, the pressure gradient may be less severe than expected for the degree of obstruction due to the development of collateral vessels. Patients can also develop lower extremity claudication or abdominal angina based on the level and location of the coarctation.^14^

Although, in classical CoA cases, transthoracic echocardiography is the screening method of choice, MAC is better diagnosed using CT angiography due to its retrothoracal–retroperitoneal location. Treatment of MAC usually entails aorto-aortic bypass of the diseased segment or endovascular angioplasty with stenting in short isolated stenosis. Evidence-based guidelines on the management of MAC during pregnancy are lacking. In a clinical case report by Kilic *et al*., undiagnosed MAC prior to conception led to superimposed pre-eclampsia at 30 weeks of gestation. The patient had severe hypertension (BP: 210/110 mmHg), resulting in an emergency caesarean section and maternal treatment with intravenous nitroglycerin. Middle aortic coarctation was diagnosed postpartum due to persistently elevated BP after placental delivery. In that case, the coarctation was not amenable to endovascular therapy and the patient declined surgical treatment.^15^ In a second case, reported by Rabstein *et al*., the patient underwent a tunnelled descending thoracic aorto-left common iliac artery bypass using a 22 cm × 9 mm Dacron graft. Following the surgical intervention, the authors reported a normal pregnancy and delivery of a full-term baby.^16^

During pregnancy, MAC can lead to an unfavourable feto-maternal haemodynamic balance. On the one hand, elevation of the arterial BP of the upper part of the body increases the maternal risk of stroke, aortic dissection, or heart failure. On the other hand, hypo-perfusion of the lower part of the body reduces uterine perfusion. Chronic uterine hypoxia and placental malperfusion are the leading cause of failed placentation. In turn, failed placentation is the pathophysiological basis for the development of pre-eclampsia^17^ and/or placental insufficiency with foetal growth restriction.^18^ The management of MAC in gestating women can be particularly challenging. Indeed, angiotensin-converting enzyme inhibitors, angiotensin receptor blockers, or direct renin inhibitors are category X and strictly contraindicated in pregnancy.^19^ Furthermore, PTA exposes the foetus to radiation and reports for CoA angioplasty and stenting during pregnancy are very limited. Thus, we decided on an interventional rather than conservative approach. The persistence of an elevated BP profile in our patient may be explained by MAC-associated global endothelial dysfunction. However, we consider that we significantly reduced the risk of adverse pregnancy outcome associated with chronic hypertension and simplified the management of any future pregnancies.

### Follow-up proposal for future pregnancies

Due to persistent BP elevation and a new abdominal stent, the patient would benefit from a multidisciplinary follow-up during pregnancy.

From a cardiologic and obstetric point of view, the main goals will be to monitor and maintain BP < 140/90 mmHg throughout pregnancy to prevent serious hypertensive pregnancy complications. For state-of the-art BP monitoring, we suggest performing 24h-ABPM at least each trimester in addition to office or home measurement. Indeed, 24h-ABPM has shown to better predict the development of severe hypertension compared with office measurement in pregnant women, and 24h-ABPM is now endorsed by the 2018 ESC guidelines for the monitoring of high-risk pregnant women with hypertension.^7^ For BP control, we suggest continued treatment with labetalol, with dose adjustment to the physiological fall in BP expected in the first half of the pregnancy. Labetalol, methyldopa, and calcium channel blockers have all shown to be safe during pregnancy and are currently the preferred choice in the treatment of moderate CHP.^7^ Regarding the cost-benefit of treating mild CHP, outdated data had suggested that BP reduction may increase the risk of small for gestational age or low birth weight.^18^ However, two recent multi-centric randomized clinical trials, Control of Hypertension in Pregnancy Study (CHIPS) and Chronic Hypertension and Pregnancy (CHAP), have shown that a tight antihypertensive control in pregnant women with mild hypertension does not restrict foetal growth. Furthermore, these two trials have shown a reduction of severe hypertension (160/110 mmHg) in thigh control groups. CHAP trial further showed a significant decrease of composite outcomes including pre-eclampsia with severe features, preterm birth, placental abruption, and perinatal death.

From an obstetrical point of view, the main goals of the management of this patient during pregnancy will be to prevent or treat superimposed pre-eclampsia and detect any alteration of the uteroplacental function leading to the development of foetal growth restriction. Laboratory monitoring of hypertensive pregnant women includes regular urine analysis to detect proteinuria, blood count, haematocrit, liver enzymes, serum creatinine, and serum uric acid and to evaluate angiogenic imbalance after 20 weeks by investigating the course of the marker placental-like growth factor (PlGF) and soluble fms-like tyrosine kinase-1 (s-Flt-1) and its ratio. More recently, these new angiogenic markers have helped to monitor placental function and allow for an early detection of placentation disorders. They also have the potential to tailor individual management based on regular foetal biometric assessment and Doppler investigations of the uteroplacental and fetoplacental compartment in the third trimester of pregnancy. In cases that manifest foetal growth restriction, PlGF and s-Flt-1 measurements offer precious help in the decision-making process of the delivery of a compromised foetus.

Given the presence of both a new stent and chronic hypertension, we suggest initiating aspirin 100 mg q.d. at the time of pregnancy diagnosis (positive pregnancy test). Between 11 and 14 gestational weeks, we suggest performing a pre-eclampsia screening test as recommended by the Fetal Medicine Foundation in London. In the case of a positive screening test, aspirin dosage should be increased to 150 mg q.d. in accordance with the results of the ASPRE trial.^20^

From a surgical point of view, the most feared complication would be restenosis or an acute stent thrombosis during pregnancy. Indeed, the physiological hyper-coagulable state induced by pregnancy may theoretically foster stent thrombus. To screen for this complication, we suggest performing ABI each trimester. As primary prevention, we suggest a daily antiplatelet prophylaxis with low-dose aspirin to be continued 4–6 weeks of postpartum, as mentioned earlier. If restenosis occurs, a repeat PTA could be performed if absolutely necessary but preferably after the fourth month of pregnancy when organogenesis is complete and the foetal thyroid is still inactive. Nevertheless, due to the abdominal location of the CoA, PTA would expose the foetus to considerable ionizing radiation with limited options to minimize radiation. Alternatively, thrombolysis could also be intended, as recombinant tissue plasminogen activator does not cross the placenta; this procedure is however associated with a significant risk of maternal and placental bleeding.^6^

## Conclusion

This case illustrates the importance of preconception screening and treatment of chronic hypertension in women who plan to become pregnant. It highlights the fact that secondary causes of hypertension should not be overlooked in this population. The value of careful cardio-obstetrical evaluation is important as it can reduce cardiovascular morbidity and mortality during and after pregnancy.

## Supplementary Material

ytae092_Supplementary_Data

## Data Availability

The data underlying this article are available in the article and in its online Supplementary material.

## Lead author biography

Dr Theo A. Meister is a junior clinician scientist; his research field focuses on alterations of the cardiac phenotype induced by particular events during pregnancy, including maternal hypertension or assisted reproductive technologies.
